# Cerebrospinal Fluid in Classical Trigeminal Neuralgia: An Exploratory Study on Candidate Biomarkers

**DOI:** 10.3390/biomedicines10050998

**Published:** 2022-04-26

**Authors:** Teodor Svedung Wettervik, Dick Folkvaljon, Torsten Gordh, Eva Freyhult, Kim Kultima, Hans Ericson, Sami Abu Hamdeh

**Affiliations:** 1 Department of Medical Sciences, Section of Neurosurgery, Uppsala University, SE-751 85 Uppsala, Sweden; d.folkvaljon@gmail.com (D.F.); hans.ericson@akademiska.se (H.E.); sami.abu.hamdeh@neuro.uu.se (S.A.H.); 2 Department of Surgical Sciences, Pain Research, Anesthesiology and Intensive Care, Uppsala University, SE-751 85 Uppsala, Sweden; torsten.gordh@surgsci.uu.se; 3 Department of Cell and Molecular Biology, Uppsala University, 75124 Uppsala, Sweden; eva.freyhult@icm.uu.se; 4 Department of Medical Sciences, Chemical Chemistry, Uppsala University, SE-751 85 Uppsala, Sweden; kim.kultima@medsci.uu.se

**Keywords:** cerebrospinal fluid, demyelination, neuroinflammation, trigeminal neuralgia

## Abstract

Trigeminal neuralgia (TN) is a severe type of facial pain. A neurovascular conflict between cranial nerve V and a nearby vessel is the main pathophysiological mechanism, but additional factors are likely necessary to elicit TN. In this study, the primary aim was to explore differences in protein expression in the cerebrospinal fluid (CSF) of TN patients in relation to controls. **Methods:** Sixteen TN patients treated with microvascular decompression and 16 control patients undergoing spinal anesthesia for urological conditions were included. Lumbar CSF was collected preoperatively for the TN patients and before spinal anesthesia for the controls. A multiplexed proximity extension analysis of 91 CSF proteins was conducted using Proseek Multiplex Development 96, including biomarkers of cell communication, cell death, neurogenesis, and inflammation **Results:** The TN patients and the controls were of similar age, sex, and burden of co-morbidities. The TN patients exhibited higher concentrations of Clec11a, LGMN, MFG-E8, and ANGPTL-4 in CSF than the controls (q < 0.05). **Conclusions:** TN patients exhibited increased CSF biomarkers indicative of peripheral demyelinating injury (Clec11a), immune tolerance and destruction of myelin (LGMN), neuronal cell death (MFG-E8), and disturbances in myelin clearance (ANGPTL-8). Our findings are hypothesis-generating for candidate biomarkers and pathophysiological processes in classical TN.

## 1. Introduction

Trigeminal neuralgia (TN) is characterized by brief and intense episodes of sharp facial pain, usually located in the V2/V3 dermatome of the trigeminal nerve (CN V) [1]. The estimated prevalence is between 0.1–0.3% [2,3] and the disease causes severe suffering for these patients with a significant reduction in the quality of life [4]. Classical TN has typically been attributed to a neurovascular conflict (NVC) between a cerebral vessel (most often the superior cerebellar artery) and CN V at the root entry zone (REZ) [5]. The NVC may induce dislocation, demyelination, and atrophy of CN V [6]. According to the ignition hypothesis, the injured, demyelinated CN V is susceptible to ephaptic transmission of innocuous somatosensory stimuli, which activate pain fibers and elicit severe facial pain in the corresponding nerve territory [6]. Still, it is evident that not all patients with NVC develop TN, since a simple neurovascular contact is also frequent in many asymptomatic cases [7,8]. It usually requires a pronounced NVC with additional morphological CN V changes such as distortion, dislocation, and atrophy, to develop classical TN [7,9]. However, it is likely that also other mechanisms than the mechanical, anatomical conflict are important in TN pathophysiology. For example, gene-related differences of the sodium channels of the axonal membrane influence their nerve transmission and affect the susceptibility to develop TN [10]. Furthermore, we recently found that TN patients exhibited elevated biomarkers of neuroinflammation and cell death in the cerebrospinal fluid (CSF) compared with controls, which normalized to the concentrations of controls after microvascular decompression (MVD) [11,12]. MVD is a surgical procedure that targets the NVC by dissecting CN V from the conflicting vessel and placing a material (e.g., Teflon) to reduce the risk of NVC recurrence, in medically refractory TN patients with radiological evidence of an NVC [13]. The role of neuroinflammation in classical TN is particularly interesting, since TN is also frequent in patients with the neuroinflammatory disease multiple sclerosis (MS) [1]. Although classical TN and MS-related TN are two distinct entities, they may share similar disease processes such as neuroinflammation, demyelination, and atrophy.

In the current study, the primary aim was to further explore potential pathophysiological mechanisms in classical TN by CSF analyses of protein biomarkers using the Proseek Multiplex Development 96 panel (Olink Bioscience, Uppsala, Sweden). The main findings were that TN patients exhibited elevated CSF biomarkers of neuroinflammation and neuronal injury.

## 2. Materials and Methods

### 2.1. Patients and Study Design

In this prospective observational study, patients referred to the Department of Neurosurgery at Uppsala University Hospital, who fulfilled the criteria for classical TN, according to the beta version of the International Classification of Headache disorders, third edition [14], and were candidates for surgical treatment with MVD between 2015 and 2017 were eligible for the study. The lumbar CSF of the TN patients was analyzed in relation to an aged-matched control group of 16 patients undergoing minor urological surgery under spinal anesthesia (University Hospital, Cluj, Romania). 

### 2.2. CSF Collection

In the TN group, CSF was collected from the day before surgery by lumbar puncture without anesthesia. In the control group of urological patients, the lumbar CSF sample was collected by free flow into a sterile tube before the spinal anesthesia was administered. The CSF samples from both groups were handled according to the same protocol and were stored in a −70 °C freezer in both groups. The CSF samples from Cluj, Romania, were shipped to Uppsala, Sweden, by professional cargo, and kept frozen at −70 °C during delivery.

### 2.3. Proximity Extension Analysis

Multiplexed proximity extension analysis (PEA) was conducted using Proseek Multiplex Development 96 including 92 protein biomarkers. The Development panel analyzed a different subset of biomarkers as compared to the Inflammation panel that was used in a previous study by our group [12]. The current biomarker panel included a wide array of biological processes such as cell communication, cell death, neurogenesis, and inflammation (Supplementary Table S1). The protein biomarker neurofascin was excluded due to technical issues with the analysis. The PEA is a technique to analyze multiple biomarkers simultaneously, requires only a small sample volume (µL), and carries a high sensitivity and specificity. The proteins are reported in arbitrary units as normalized protein expression (NPX).

The procedure has been described in detail in a previous study by our group [12]. One µL CSF serum was mixed with a 3-µL incubation mix and incubated at 8 °C overnight. A 96-mL extension mix based on PEA enzyme and PCR reagents were added, incubated for 5 min at room temperature, and the plate was then transferred to a thermal cycler for an extension reaction followed by 17 cycles of DNA amplification. A 96.96 Dynamic Array IFC (Fluidigm, South San Francisco, FL, CA) was prepared and primed. In a new plate, a 2.7 µL of sample mixture was mixed with a 7.2-µL detection mix, from which 5 µL was loaded into the right side of the primed 96.96 Dynamic Array IFC.

Five µL of the primer pairs were loaded into the left side of the 96.96 Dynamic Array IFC, and the protein expression program was run in a Fluidigm Biomark reader.

### 2.4. Statistical Analysis

The primary aim of this study was to explore potential CSF biomarkers in TN in relation to a control group. The categorical and ordinal/continuous variables were presented as numbers (proportions) and medians (interquartile range (IQR)), respectively. Differences in demographic variables between the TN patients and the controls were assessed with the Chi-square or Mann–Whitney U-test, depending on the type of data. A principal component analysis (PCA) was performed to investigate if there were global differences between TN and controls. Only biomarkers with less than 20% missing values were included.

Differences in CSF biomarker concentrations between TN and controls were assessed with linear regression analyses for each biomarker, adjusting for age and sex. To account for multiple comparisons, the false-discovery rate (FDR; Benjamini–Hochberg; q-values) procedure was used. Only biomarkers with a q-value < 0.05 were considered statistically significant. Furthermore, a multivariate partial least squares discriminant analysis (PLS-DA) classification model was trained to investigate if biomarker concentrations can be used to distinguish between TN and controls. Only biomarkers with less than 20% missing values were included in the model. The predictive ability of the PLS-DA models was evaluated by 10 5-fold cross-validations. The variable importance (VIP) was reported for the analyzed biomarkers.

### 2.5. Ethics

The study was conducted in accordance with the Helsinki declaration and its later amendments. The study was approved by the Regional Research Ethics Committee in Uppsala (Approval number 2014-178, date: 24 June 2014) for the TN patients and by the Institutional ethics committee of University of Medicine and Pharmacy, Cluj-Napoca, Romania (Approval number: Not applicable for this committee. Date: 20 May 2010) for the controls. Written informed consent was obtained by all study participants.

## 3. Results

### Patients

Demographic details of the TN patients and the controls are described in Table 1. The TN patients and the controls were of a similar age (median 66 (IQR 55–69) vs. 67 (IQR 52–72) years) and female/male ratio (7/9 (44/56%) vs. 6/10 (38/63%)). Both groups also exhibited similar body mass index (BMI) and American Society of Anesthesiologist (ASA) grade.

Of the 91 biomarkers measured using Proseek Multiplex Development 96, 20 were excluded from the analyses since more than 20% of the patients had missing values (under the limit of detection).

**Figure 1:**
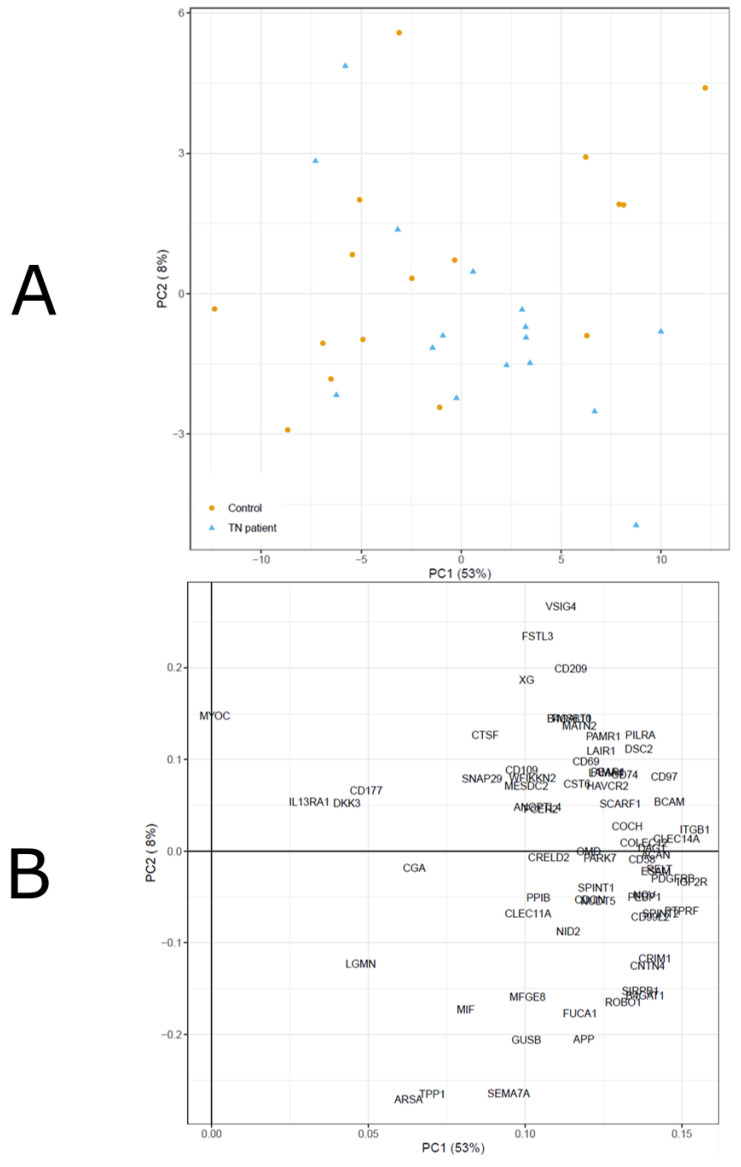
(**A**,**B**). CSF biomarkers in TN patients and controls—a PCA analysis.

The PCA analysis of the CSF biomarker concentrations showed no clear differences between TN patients and controls (Figure 1).

The PCA analysis (Figure 1A) and the corresponding loadings (Figure 1B) of the CSF biomarker concentrations showed no clear differences between TN patients and controls.

To investigate if biomarker concentrations differed between TN patients and controls, a linear regression analysis was performed, adjusted for age and sex (Supplementary Table S2). Out of the 71 CSF biomarkers, four were significantly (q < 0.05) elevated in the TN patients (Table 2). These CSF biomarkers were Clec11a, LGMN, MFG-E8, and ANGPTL-4. None of the remaining 67 showed any tendency to differ between TN and controls.

**Figure 2:**
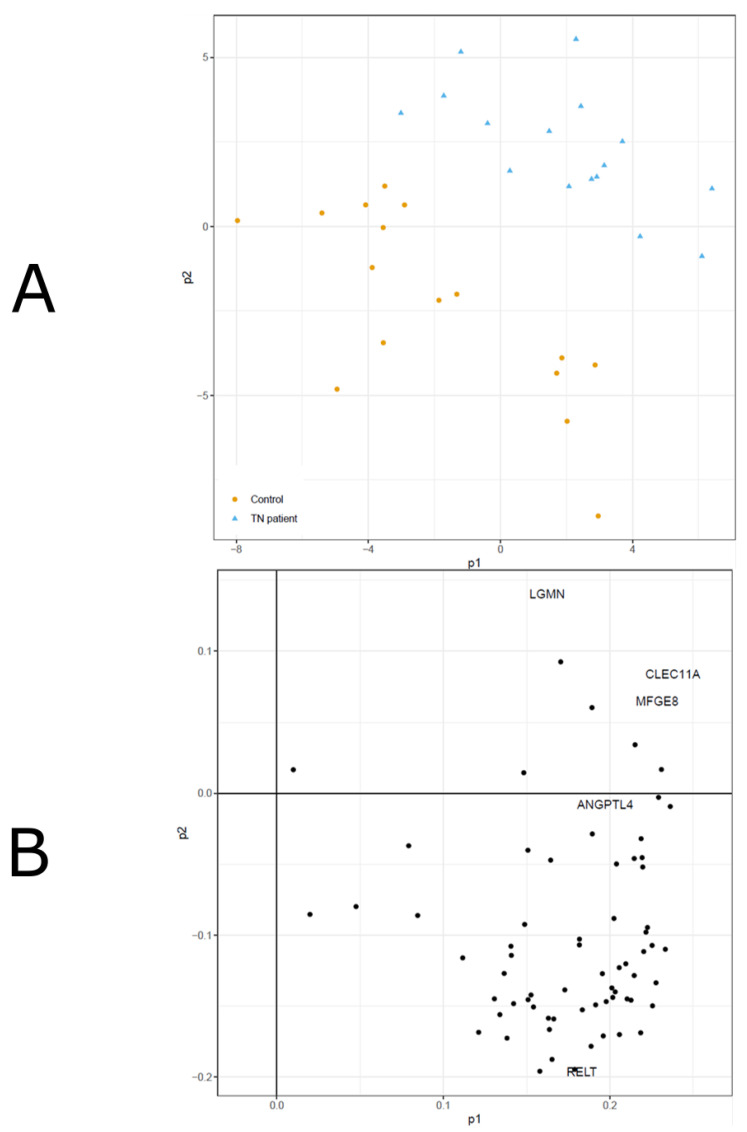
(**A**,**B**). CSF biomarkers in TN patients and controls—a PLS-DA analysis. A PLS-DA model (2A) and the corresponding loadings (2B) of CSF biomarkers in TN patients and controls are demonstrated. The model was trained to separate between TN and controls. The model showed good performance (mean error rate = 0.058) in separating between the two groups as evaluated in the cross-validation procedure. In the loadings plot, all proteins with VIP > 1.3 are named, and the remaining proteins are only shown as dots.

A PLS-DA model was trained to separate between TN and controls (Figure 2). The model showed good performance (mean error rate = 0.058) in separating between the two groups as evaluated in the cross-validation procedure.

**Figure 3:**
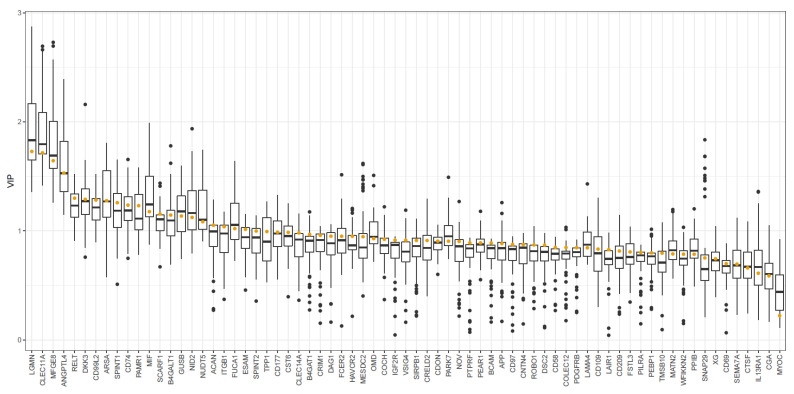
CSF biomarkers in TN patients and controls—a PLS-DA VIP analysis. The figure demonstrates the VIP values for clinical and biomarker variables in the PLS-DA model predicting patient or control CSF samples based on PEA data. The yellow dots represent the VIP for the full model (based on all data). The boxplots represent the VIP for the cross-validated models.

The four most important proteins in the model all had a mean VIP score >1.5 (Figure 3), and were the same proteins (LGMN, Clec11a, MFG-E8, and ANGPTL-4) as found significantly elevated in the linear models.

**Figure 4:**
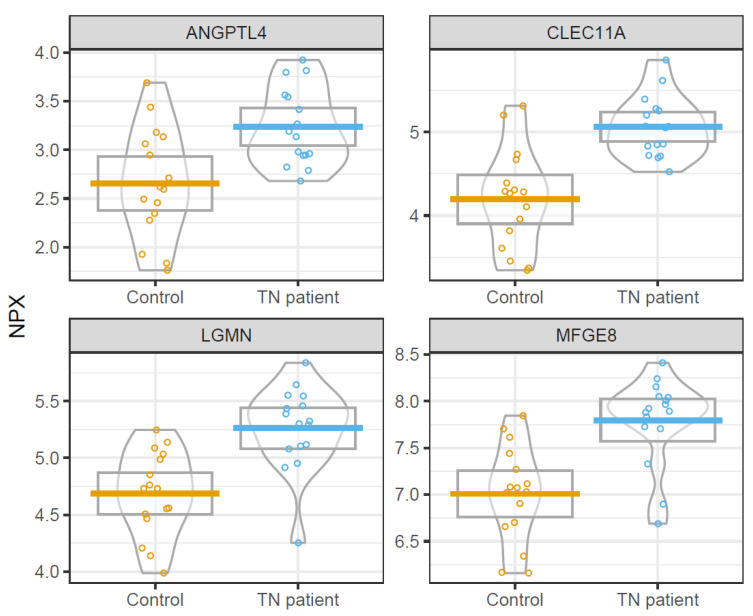
Elevated CSF biomarkers in TN patients. The figure demonstrates boxplots of ANGPTL-4, Clec11a, LGMN, and MFG-E8 in TN patients and controls.

Figure 4 demonstrates differences in concentration of these four proteins between TN patients and controls.

## 4. Discussion

In this exploratory study of CSF biomarkers, we found that TN patients exhibited different protein concentration profiles as compared to controls. Particularly, Clec11a, LGMN, MFG-E8, and ANGPTL-4 were significantly higher in CSF in TN patients. These biomarkers are involved in neuroinflammation and myelin turnover and may reflect the demyelination and nerve atrophy that is commonly seen in TN.

Clec11a is generally known as a glycoprotein involved in hematopoiesis and osteoblast maturation [15]. There is a paucity of studies on Clec11a in CNS disorders. However, it seems that the CSF content of Clec11a is elevated in chronic inflammatory demyelinating disease (CIDP) compared to both MS and neurological conditions not primarily characterized by neuroinflammation [16]. The authors hypothesized that, considering the connection between Clec11a and hematopoiesis, Clec11a might particularly drive inflammation in peripheral nerves. This is interesting since the NVC in classical TN is located in the REZ and may affect both the central and peripheral component of the nerve. Elevated Clec11a in TN might hence reflect inflammatory injury to the peripheral part of CN V. Furthermore, in a study by Stein et al., blood levels of Clec11 gradually increased with time in patients with chronic spinal cord injury, possibly reflecting chronic nerve degeneration [17]. Altogether, Clec11a may reflect chronic CN V demyelination and nerve degeneration.

LGMN, also known as osteolectin, is a protease located in the endoplasmatic reticulum, golgi apparatus, and the lysosome. In pathological conditions, it may be translocated to the cytosol and extracellular compartment [18]. One major function is to regulate the lysosomal processing of proteins that are ultimately presented at the major histocompatibility complex II (MHC-II). Particularly, increased LGMN is associated with increased degradation of myelin based protein (MBP) in antigen-presenting immune cells. This can predispose for decreased MBP immune tolerance, leading to increased autoimmune T-cell activity and destruction of MBP [19]. Increased LGMN has also been found in active and chronic lesions of white matter in humans, suggesting ongoing neuroinflammation [20]. Hence, elevated LGMN may reflect an increased propensity to develop immune reactions towards the myelin and could indicate ongoing inflammation in the white matter.

MFG-E8 is a multifunctional glycoprotein that is mainly present in lactating mammary glands, but also in many other organs such as the heart, lungs, vessels, and the brain [21]. One function of MFG-E8 is to enhance phagocytosis of apoptotic cells, which also has an anti-inflammatory effect since it reduces the pro-inflammatory signaling cascade. Previous studies suggest that MFG-E8 mediates phagocytic neuronal cell death during neuroinflammation [22] and higher MFG-E8 in the TN patients may hence reflect trigeminal degeneration. Furthermore, we have previously found that the tumor necrosis factor (TNF)-β is elevated in CSF in TN patients [12]. TNF-β is known to contribute to the development of tertiary lymphoid organs. We speculated if this reflects the development of arachnoiditis that is often seen in association with CN V in TN. Interestingly, MFG-E8 is produced by these tertiary lymphoid organs [23] and elevated MFG-E8 might possibly be a reflection of such arachnoiditis. However, it should also be mentioned that MFG-E8 may be neuroprotective in some CNS diseases. In Alzheimer’s disease, MFG-E8 increases beta-amyloid clearance and, in cerebral ischemia, it ameliorates neuroinflammation [21,24]. In addition, another role for MFG-E8 is in vascular ageing, as it promotes the development of atherosclerosis [21]. Classical TN typically occurs in older patients and may be associated with vascular ageing and degeneration, as indicated by a higher MFG-E8.

ANGPTL-4 is a glycoprotein [25,26] that is involved in angiogenesis, lipid metabolism, and inflammation [27]. Particularly in CNS diseases, increased ANGPTL-4 reduces macrophage and microglia clearance of damaged myelin [28,29]. In MS, ANGPTL-4 is reduced in active lesions, which is considered favorable since it increases clearance of damaged myelin and enhances remyelination and recovery [29]. Higher ANGPTL-4 in TN may therefore indicate disturbed myelin clearance with reduced chances of remyelination [30].

Altogether, our findings indicate increased neuroinflammation and disturbances in myelin turnover in TN. Particularly, many of these proteins have been studied in MS. This is interesting considering the relatively high burden of TN in MS patients.^1^ Although TN is usually explained by demyelinating lesions of the trigeminal pathways in the pons in MS, an NVC at the REZ is occasionally present and has even been suggested to drive demyelination in some of these MS patients [31,32]. Hence, despite the fact that patients with classical and MS-related TN are considered two distinct disease entities, they may exhibit both similar and different underlying pathophysiological mechanisms. Since NVC does not always lead to development of TN, additional factors may be necessary. Based on our findings, patients with classical TN exhibited CSF biomarkers indicative of decreased immune tolerance towards MBP, and these patients may have an increased propensity towards neuroinflammatory reactions of CN V. Similar to MS, there may also be increased neuroinflammatory phagocytosis leading to atrophy of CN V in TN. However, in contrast to MS, we also found that CSF biomarkers related to demyelination of the peripheral nervous system were elevated in the patients with classical TN. The TN patients also exhibited increased ANGPTL-4, suggesting a reduced capacity to clear damaged myelin. These findings are hypothesis-generating for potential pathophysiological pathways in classical TN and highlight candidate biomarkers that may be relevant for future research efforts.

Lastly, our findings should also be viewed in light of important contributions regarding CSF analyses in TN patients. Others have found increases in substance P, calcitonin gene-related peptide (CGRP), and vasoactive intestinal peptide (VIP) as well as reductions in β-endorphin, serotonin, and dopamine in CSF [33,34], reflecting additional neurochemical pathophysiological mechanisms of relevance in this disease.

### Methodological Considerations

It is evident that NVC is not sufficient to elicit classical TN, and there is a need to elucidate further pathophysiological mechanisms. There is so far a paucity of studies assessing CSF biomarkers that may be involved in these processes [12]. This observational, prospective study is therefore an important exploratory contribution to this field. However, there are some limitations. The relatively low number of patients in both the TN and the control group increases the risk of false positive findings. At the same time, many of the studied proteins were functionally related and statistical methods were applied to adjust for multiple comparisons. We therefore think reasonably safe conclusions can be made. Furthermore, it is a challenge to select and obtain CSF from an appropriate control group. In this study, we chose to include individuals of similar age, sex, and health (ASA) who also awaited surgery, but for urological conditions and from another country (Sweden vs. Romania). The controls were chosen to mimic the pre-surgery situation experienced by the TN patients. However, we cannot exclude that factors that were not accounted for between the groups affected the results to some extent. Furthermore, CSF samples were handled according to the same protocol for both the TN and control patients, although some minor differences could have occurred. Another limitation is that we only included TN patients at a stage with medically refractory pain, which predisposed for the indication to proceed with MVD surgery. It is possible that a different biomarker pattern would occur at another stage of the disease.

## 5. Conclusions

TN patients exhibited increased CSF biomarkers indicative of peripheral demyelinating injury (Clec11a), immune tolerance and destruction of myelin (LGMN), neuronal cell death (MFG-E8), and disturbances in myelin clearance (ANGPTL-8). Our findings are hypothesis-generating for candidate biomarkers and pathophysiological processes in classical TN.

## Figures and Tables

**Table 1:** Demographic data in TN patients and controls.

Variables	TN	Controls	*p*-Value
Patients, n	16	16	
Age (years), median (IQR)	66 (55–69)	67 (52–72)	1.00
Sex (female/male), n (%)	7/9 (44/56%)	6/10 (38/63%)	0.72
BMI, median (IQR)	28 (24–31)	28 (24–31)	0.93
Tobacco use, n (%)	4 (25%)	8 (50%)	0.14
Hypertension, n (%)	8 (50%)	9 (56%)	0.72
Cardiovascular morbidity, n (%)	2 (13%)	5 (31%)	0.22
Ischemic heart disease, n (%)	1 (6%)	3 (19%)	
Stroke, n (%)	1 (6%)	1 (6%)	
Peripheral, n (%)	0 (0%)	1 (6%)	
Diabetes mellitus, n (%)	0 (0%)	3 (19%)	0.07
Pain other than TN, n (%)	4 (25%)	5 (31%)	0.69
Fibromyalgia, n (%)	1 (6%)	0 (0%)	
Osteoarthtritis, n (%)	2 (13%)	2 (13%)	
Lower back pain, n (%)	1 (6%)	2 (13%)	
Rheumatoid arthritis, n (%)	1 (6%)	1 (6%)	
ASA grade			0.83
1, n (%)	2 (13%)	2 (13%)	
2, n (%)	13 (81%)	12 (75%)	
3, n (%)	1 (6%)	2 (13%)	

Lumbar CSF—in TN patients and controls.

**Table 2:** Differences in spinal CSF biomarker concentrations between TN patients and controls.

Biomarker	Coefficient	*p* (lm)	q (lm)
*Clec11a*	0.85	5.46 × 10^−5^	0.0037
*LGMN*	0.59	0.00015	0.0037
*MFG-E8*	0.79	0.00016	0.0037
*ANGPTL-4*	0.60	0.00097	0.017

The table demonstrates the significant CSF biomarkers that differed between TN patients and controls. Each biomarker was analyzed using linear regression, adjusting for age and sex. The first column is the regression coefficient (positive value if higher biomarker concentration in the TN group), second column is the p- and third column contain q-values (FDR (Benjamini–Hochberg) adjusted *p*-values). Only proteins that were significant at the 5% FDR level are included in the table.

## Data Availability

The data are available from the authors upon reasonable request.

## Supplementary Materials

The following supporting information can be downloaded at: https://www.mdpi.com/article/10.3390/biomedicines10050998/s1, Table S1: Analyzed CSF biomarkers with the Proseek Multiplex Development 96; Table S2: Differences in spinal CSF biomarker concentrations between TN patients and controls–the entire analysis.
