# Supplementary material for: Cerebrospinal Fluid in Classical Trigeminal Neuralgia: An Exploratory Study on Candidate Biomarkers
Source: Biomedicines. 2022 Apr 26;10(5):998. doi: 10.3390/biomedicines10050998 (PMC9138315; doi:10.3390/biomedicines10050998)
Supplement: Supplementary file 1 [file biomedicines-10-00998-s001.zip › biomedicines-1695388-supplementary.pdf]

Supplementary Table S1. Analyzed CSF biomarkers with the Proseek Multiplex Development 96

**Biomarkers**
ADP-ribosyl cyclase/cyclic ADP-ribose hydrolase 2 (BST1)
ADP-sugar pyrophosphatase (NUDT5)
Aggrecan core protein (ACAN)
Amyloid beta A4 protein (APP)
Angiopoietin-related protein 4 (ANGPTL4)
Arylsulfatase A (ARSA)
Basal cell adhesion molecule (BCAM)
Beta-1,4-galactosyltransferase 1 (B4GALT1)
Beta-1,4-glucuronyltransferase 1 (B4GAT1)
Beta-glucuronidase (GUSB)
Beta-microseminoprotein (MSMB)
C-C motif chemokine 21 (CCL21)
C-type lectin domain family 11 member A (CLEC11A)
C-type lectin domain family 14 member A (CLEC14A)
Carbonic anhydrase 2 (CA2)
Carbonic anhydrase 6 (CA6)
Cathepsin F (CTSF)
Cation-independent mannose-6-phosphate receptor (IGF2R)
CCN family member 5 (CCN5)
CD109 antigen (CD109)
CD177 antigen (CD177)
CD209 antigen (CD209)
CD97 antigen (CD97)
CD99 antigen-like protein 2 (CD99L2)
Cell adhesion molecule-related/down-regulated by oncogenes (CDON)
CMRF35-like molecule 9 (CD300LG)
Cochlin (COCH)
Collectin-12 (COLEC12)
Contactin-4 (CNTN4)
Corticotropin-releasing factor-binding protein (CRHBP)
Cystatin-M (CST6)
Cysteine-rich motor neuron 1 protein (CRIM1)
Cysteine-rich with EGF-like domain protein 2 (CRELD2)
Cytosolic phospholipase A2 (PLA2G4A)
Desmocollin-2 (DSC2)
Dickkopf-related protein 3 (DKK3)
Dystroglycan (DAG1)
Early activation antigen CD69 (CD69)
Endothelial cell-selective adhesion molecule (ESAM)
Fc receptor-like protein 5 (FCRL5)
Flavin reductase NADPH (BLVRB)
Follistatin-related protein 3 (FSTL3)
Galactoside 3(4)-L-fucosyltransferase,Alpha-(1,3)-fucosyltransferase 5 (FUT3/5)
Glycoprotein hormones alpha chain (CGA)
Glycoprotein Xg (XG)
Hepatitis A virus cellular receptor 2 (HAVCR2)

HLA class II histocompatibility antigen gamma chain (CD74)
Inactive serine protease PAMR1 (PAMR1)
Inhibin beta C chain (INHBC)
Integrin alpha-5 (ITGA5)
Integrin beta-1 (ITGB1)
Interleukin-13 receptor subunit alpha-1 (IL13RA1)
Kunitz-type protease inhibitor 1 (SPINT1)
Kunitz-type protease inhibitor 2 (SPINT2)
Lactadherin (MFGE8)
Laminin subunit alpha-4 (LAMA4)
LDLR chaperone MESD (MESDC2)
Legumain (LGMN)
Leukocyte-associated immunoglobulin-like receptor 1 (LAIR1)
Low affinity immunoglobulin epsilon Fc receptor (FCER2)
Lymphocyte function-associated antigen 3 (CD58)
Macrophage migration inhibitory factor (MIF)
Matrilin-2 (MATN2)
Myocilin (MYOC)
Nidogen-2 (NID2)
Osteomodulin (OMD)
Paired immunoglobulin-like type 2 receptor alpha (PILRA)
Peptidyl-prolyl cis-trans isomerase B (PPIB)
Phosphatidylethanolamine-binding protein 1 (PEBP1)
Platelet endothelial aggregation receptor 1 (PEAR1)
Platelet-derived growth factor receptor beta (PDGFRB)
Protein deglycase DJ-1 (PARK7)
Protein disulfide-isomerase (P4HB)
Protein NOV homolog (NOV)
Receptor-type tyrosine-protein phosphatase F (PTPRF)
Roundabout homolog 1 (ROBO1)
Scavenger receptor class F member 1 (SCARF1)
Semaphorin-7A (SEMA7A)
Serine protease HTRA2, mitochondrial (HTRA2)
Serine protease inhibitor Kazal-type 1 (SPINK1)
Serine protease inhibitor Kazal-type 5 (SPINK5)
Signal-regulatory protein beta-1 (SIRPB1)
Stress-induced-phosphoprotein 1 (STIP1)
Synaptosomal-associated protein 29 (SNAP29)
Thymosin beta-10 (TMSB10)
Tissue alpha-L-fucosidase (FUCA1)
Tripeptidyl-peptidase 1 (TPP1)
Tumor necrosis factor receptor superfamily member 19L (RELT)
Tyrosine-protein phosphatase non-receptor type 6 (PTPN6)
V-set and immunoglobulin domain-containing protein 4 (VSIG4)
WAP, Kazal, immunoglobulin, Kunitz and NTR domain-containing protein 2 (WFIKKN2)

Supplementary Table S2. Differences in spinal CSF biomarker concentrations between TN patients and controls – the entire linear regression analysis

Biomarker	Coefficient	p (lm)	q (lm)
C-type lectin domain family 11 member A (Clec11a)	0.85	$5.46e^{-05}$	0.0037
Legumain (LGMN)	0.59	0.00015	0.0037
Lactadherin (MFG-E8)	0.79	0.00016	0.0037
Angiopoietin-related protein 4 (ANGPTL-4)	0.60	0.00097	0.018
Nidogen-2 (NID2)	0.26	0.015	0.19
Macrophage migration inhibitory factor (MIF)	0.86	0.016	0.19
Arylsulfatase A (ARSA)	0.31	0.028	0.28
Beta-glucuronidase (GUSB)	0.23	0.038	0.30
Osteomodulin (OMD)	0.30	0.042	0.30
Tissue alpha-L-fucosidase (FUCA1)	0.33	0.042	0.30
ADP-sugar pyrophosphatase (NUDT5)	0.35	0.051	0.33
Protein deglycase DJ-1 (PARK7)	0.39	0.056	0.33
Laminin subunit alpha-4 (LAMA4)	0.18	0.069	0.38
Matrilin-2 (MATN2)	0.21	0.090	0.41
Interleukin-13 receptor subunit alpha-1 (IL13RA1)	-0.10	0.092	0.41
Peptidyl-prolyl cis-trans isomerase B (PPIB)	0.24	0.092	0.42
Platelet endothelial aggregation receptor 1 (PEAR1)	0.15	0.14	0.57
Beta-1,4-galactosyltransferase 1 (B4GALT1)	-0.15	0.16	0.61
Dickkopf-related protein 3 (DKK3)	-0.07	0.18	0.66
HLA class II histocompatibility antigen gamma chain (CD74)	-0.22	0.19	0.67
Platelet-derived growth factor receptor beta (PDGFRB)	0.16	0.25	0.82
Cell adhesion molecule-related/down-regulated by oncogenes (CDON)	0.09	0.26	0.82
Phosphatidylethanolamine-binding protein 1 (PEBP1)	0.22	0.26	0.82
Paired immunoglobulin-like type 2 receptor alpha (PILRA)	0.11	0.41	0.99
Tumor necrosis factor receptor superfamily member 19L (RELT)	-0.13	0.42	0.99
Cathepsin F (CTSF)	0.12	0.42	0.99
CD99 antigen-like protein 2 (CD99L2)	-0.08	0.44	0.99
Lymphocyte function-associated antigen 3 (CD58)	0.07	0.46	0.99

Cation-independent mannose-6-phosphate receptor (IGF2R)	0.09	0.48	0.99
Receptor-type tyrosine-protein phosphatase F (PTPRF)	0.11	0.48	0.99
Collectin-12 (COLEC12)	0.08	0.49	0.99
Inactive serine protease PAMR1 (PAMR1)	-0.09	0.50	0.99
Tripeptidyl-peptidase 1 (TPP1)	0.08	0.50	0.99
Basal cell adhesion molecule (BCAM)	0.10	0.51	0.99
Scavenger receptor class F member 1 (SCARF1)	-0.11	0.51	0.99
Early activation antigen CD69 (CD69)	0.09	0.53	0.99
Cysteine-rich motor neuron 1 protein (CRIM1)	0.07	0.55	0.99
Semaphorin-7A (SEMA7A)	0.08	0.56	0.99
Dystroglycan (DAG1)	0.07	0.57	0.99
CD97 antigen (CD97)	0.08	0.59	0.99
Desmocollin-2 (DSC2)	0.08	0.61	0.99
CD177 antigen (CD177)	-0.20	0.64	0.99
Cysteine-rich with EGF-like domain protein 2 (CRELD2)	-0.07	0.68	0.99
Signal-regulatory protein beta-1 (SIRPB1)	0.07	0.68	0.99
Glycoprotein Xg (XG)	0.07	0.68	0.99
Leukocyte-associated immunoglobulin-like receptor 1 (LAIR1)	0.06	0.70	0.99
V-set and immunoglobulin domain-containing protein 4 (VSIG4)	0.09	0.71	0.99
Contactin-4 (CNTN4)	0.06	0.73	0.99
Glycoprotein hormones alpha chain (CGA)	-0.10	0.74	0.99
Thymosin beta-10 (TMSB10)	0.10	0.74	0.99
LDLR chaperone MESD (MESDC2)	-0.04	0.76	0.99
Low affinity immunoglobulin epsilon Fc receptor (FCER2)	-0.08	0.76	0.99
WAP, Kazal, immunoglobulin, Kunitz and NTR domain-containing protein 2 (WFIKKN2)	0.06	0.77	0.99
Roundabout homolog 1 (ROBO1)	0.05	0.78	0.99
Follistatin-related protein 3 (FSTL3)	-0.04	0.79	0.99
Synaptosomal-associated protein 29 (SNAP29)	-0.04	0.80	0.99
Cystatin-M (CST6)	-0.04	0.81	0.99
Kunitz-type protease inhibitor 1 (SPINT1)	-0.02	0.82	0.99
Aggrecan core protein (ACAN)	0.03	0.86	0.99
Amyloid beta A4 protein (APP)	0.01	0.89	0.99

C-type lectin domain family 14 member A (CLEC14A)	0.02	0.89	0.99
Endothelial cell-selective adhesion molecule (ESAM)	-0.02	0.90	0.99
Protein NOV homolog (NOV)	0.01	0.91	0.99
CD209 antigen (CD209)	0.02	0.91	0.99
Integrin beta-1 (ITGB1)	0.01	0.92	0.99
Hepatitis A virus cellular receptor 2 (HAVCR2)	-0.02	0.92	0.99
Cochlin (COCH)	-0.01	0.97	0.99
Kunitz-type protease inhibitor 2 (SPINT2)	0.01	0.97	0.99
CD109 antigen (CD109)	-0.004	0.97	0.99
Beta-1,4-glucuronyltransferase 1 (B4GAT1)	0.002	0.98	0.99
Myocilin (MYOC)	0.003	0.99	0.99

The following 20 proteins were excluded from the analyses since more than 20% had missing values (values under the limit of detection): ADP-ribosyl cyclase/cyclic ADP-ribose hydrolase 2 (BST1), Beta-microseminoprotein (MSMB), C-C motif chemokine 21 (CCL21), Carbonic anhydrase 2 (CA2), Carbonic anhydrase 6 (CA6), CCN family member 5 (CCN5), CMRF35-like molecule 9 (CD300LG), Corticotropin-releasing factor-binding protein (CRHBP), Cytosolic phospholipase A2 (PLA2G4A), Fc receptor-like protein 5 (FCRL5), Flavin reductase NADPH (BLVRB), Galactoside 3(4)-L-fucosyltransferase,Alpha-(1,3)-fucosyltransferase 5 (FUT3/5), Inhibin beta C chain (INHBC), Integrin alpha-5 (ITGA5), Protein disulfide-isomerase (P4HB), Serine protease HTRA2, mitochondrial (HTRA2), Serine protease inhibitor Kazal-type 1 (SPINK1), Serine protease inhibitor Kazal-type 5 (SPINK5), Stress-induced-phosphoprotein 1 (STIP1), and Tyrosine-protein phosphatase non-receptor type 6 (PTPN6)
